# Treatment strategy and outcomes in locally advanced head and neck squamous cell carcinoma: a nationwide retrospective cohort study (KCSG HN13–01)

**DOI:** 10.1186/s12885-020-07297-z

**Published:** 2020-08-27

**Authors:** Yun-Gyoo Lee, Eun Joo Kang, Bhumsuk Keam, Jin-Hyuk Choi, Jin-Soo Kim, Keon Uk Park, Kyoung Eun Lee, Jung Hye Kwon, Keun-Wook Lee, Min Kyoung Kim, Hee Kyung Ahn, Seong Hoon Shin, Hye Ryun Kim, Sung-Bae Kim, Hwan Jung Yun

**Affiliations:** 1 Department of Internal Medicine, Kangbuk Samsung Hospital, Sungkyunkwan University School of Medicine, Seoul, Republic of Korea; 2 Department of Internal Medicine, Korea University Guro Hospital, Seoul, Republic of Korea; 3 Department of Internal Medicine, Seoul National University Hospital, 101 Daehak-ro, Jongno-gu, Seoul, 03080 Republic of Korea; 4 Department of Hematology-Oncology, Ajou University Hospital, Suwon, Republic of Korea; 5 Department of Internal Medicine, SMG-SNU Boramae Medical Center, Seoul, Republic of Korea; 6 Department of Hemato-Oncology, Keimyung University Dongsan Medical Center, Daegu, Republic of Korea; 7 Department of Hematology and Oncology, Ewha Womans University Hospital, Seoul, Republic of Korea; 8 Department of Internal Medicine, Hallym University College of Medicine, Kangdong Sacred Heart Hospital, Seoul, Republic of Korea; 9 Department of Internal Medicine, Seoul National University Bundang Hospital, Seongnam, Republic of Korea; 10 Department of Hematology-Oncology, Yeungnam University Medical Center, Daegu, Republic of Korea; 11 Department of Internal Medicine, Gachon University Gil Medical Center, Incheon, Republic of Korea; 12 Department of Internal Medicine, Kosin University Gospel Hospital, Busan, Republic of Korea; 13 Department of Internal Medicine, Yonsei Cancer Center, Yonsei University College of Medicine, Seoul, Republic of Korea; 14 Department of Internal Medicine, Asan Medical Center, University of Ulsan College of Medicine, Seoul, Republic of Korea; 15 Department of Internal Medicine, Chungnam National University Hospital, 282 Munhwa-ro, Jung-gu, Daejeon, 35015 Republic of Korea

**Keywords:** Locally advanced head and neck cancer, Squamous cell carcinoma, Multidisciplinary treatment, Strategy

## Abstract

**Background:**

By investigating treatment patterns and outcomes in locally advanced head and neck squamous cell carcinoma (LA-HNSCC), we aimed at providing valuable insights into the optimal therapeutic strategy for physicians in real-world practice.

**Methods:**

This is a multi-institutional study enrolled the patients with stage III to IVB LA-HNSCC, except for nasopharyngeal carcinoma, from 2004 to 2015 in thirteen referral hospitals capable of multidisciplinary care.

**Results:**

A total of 445 LA-HNSCC patients were analyzed. The median age was 61 years (range, 24–89). The primary tumor location was the oropharynx in 191 (43%), oral cavity in 106 (24%), hypopharynx in 64 (14%), larynx in 57 (13%) and other sites in 27 (6%). The most common stage was T2 in 172 (39%), and N2 in 245 (55%). Based on treatment intents, 229 (52%) of the patients received definitive concurrent chemoradiotherapy (CCRT) and 187 (42%) underwent surgery. Approximately 158 (36%) of the study population received induction chemotherapy (IC). Taken together, 385 (87%) of the patients underwent combined therapeutic modalities. The regimen for definitive CCRT was weekly cisplatin in 58%, 3-weekly cisplatin in 28% and cetuximab in 3%. The preferred regimen for IC was docetaxel with cisplatin in 49%, and docetaxel, cisplatin plus fluorouracil in 27%. With a median follow-up of 39 months, one-year and two-year survival rates were 89 and 80%, respectively. Overall survival was not significantly different between CCRT and surgery group (*p* = 0.620).

**Conclusions:**

In patients with LA-HNSCC, the majority of patients received combined therapeutic modalities. Definitive CCRT, IC then definitive CCRT, and surgery followed by adjuvant CCRT or radiotherapy are the preferred multidisciplinary strategies in real-world practice.

## Background

Head and neck squamous cell carcinoma (HNSCC) and its associated variants originate from multiple anatomic subsites in the oral cavity, oropharynx, hypopharynx and larynx. Given the heterogeneous biology of HNSCC at each subsite, treatments are complex. Generally, the primary tumor location, stage of tumor and lymph node, and pathologic characteristics guide specialized treatments including surgical procedures, radiotherapy, and/or systemic chemotherapy [1].

Around 40% of patients with HNSCC present with limited or early-stage disease, in which treatment is ordinarily single modality, either surgery or radiotherapy [2, 3]. The locally advanced (LA) HNSCC comprises the remaining 60% of patients, whom multidisciplinary modal therapy is generally recommended with either surgery followed by postoperative radiotherapy or chemoradiotherapy (CCRT), or definitive CCRT [1, 3]. Despite decades of research in the area of LA-HNSCC treatment, the clinical significance of induction chemotherapy (IC) has not been conclusive [4]. Regarding multimodal approaches for HNSCC treatment, therapeutic strategies in clinical practice depend on a multidisciplinary team approach at each hospital [1, 5]. The most effective treatment modality has yet to be established.

We describe the real-world patterns for the initial treatment of LA-HNSCC in a large nationwide cohort treated with multidisciplinary treatment modalities. By studying this population, in which patients received multidisciplinary treatment, we aim to provide valuable insights regarding the optimal therapeutic strategy for physicians.

## Methods

### Patients

This study enrolled 445 patients who were pathologically confirmed with LA-HNSCC between January 2005 and December 2015 at 13 tertiary referral hospitals located in the Republic of Korea. All the participating hospitals have their own multidisciplinary team for head and neck cancer with specialists.

LA-HNSCC was defined as clinical stage III to IVB based on the 7th edition of the American Joint Committee on Cancer [6]. Adults patients aged 20 years or older with primary squamous cell carcinoma of oropharynx, hypopharynx, larynx, oral cavity, or nasal cavity were included for analysis. Patients with biopsy-proven squamous cell carcinoma at the cervical lymph node without known origin were regarded to be of head and neck origin and were also included in this study. HPV positivity based on the results from either HPV DNA by real-time PCR or p16 expression by immunohistochemistry, depending on availability in each participating institution.

We excluded patients with nasopharyngeal cancer which differs from other HNSCC in its epidemiology, pathology, natural history and treatment, patients with distant metastasis at initial diagnosis and patients with a previous secondary malignancy diagnosed within 3 years of HNSCC diagnosis. The Institutional Review Board for main hospital (IRB-H-1304-089-481) and each participating hospital approved this study. Medical records were retrospectively reviewed for patients who were diagnosed with LA-HNSCC.

### Multidisciplinary treatment

In principle, all patients were treated according to specific treatment protocols established at each participating hospital. The treatment modality, including surgery, chemotherapy, and radiotherapy, was decided according to a multidisciplinary team approach of each hospital. When the opinions disagreed between each discipline, the agreed recommendations of the multidisciplinary care team were followed. IC is defined as chemotherapy which facilitates subsequent local therapy such as definitive CCRT or surgery. Inadequate treatment group was defined as patients who did not receive subsequent definitive treatment after diagnosis because of patient’s refusal or intolerance. All imaging studies, including MRI or CT of the head and neck, were assessed, as well as chest CT, abdominal CT, brain MRI, or positron emission tomography/CT scans where available, obtained when there were specific symptoms or clinical suspicion. Follow-up imaging was performed based on the protocol of each hospital.

### Study outcomes

The primary outcome was to identify treatment patterns that are being performed in real-world practice for the treatment of LA-HNSCC. The secondary outcome was to compare progression-free survival (PFS) and overall survival (OS) by treatment strategy and/or primary site. PFS was defined as time from diagnostic date of HNSCC until disease recurrence, progression by RECIST criteria or death of any cause. OS was defined as time from date of diagnosis to death, regardless of cause.

### Statistical analysis

Chi-square tests and independent t-tests were used to compare categorical and continuous variables between groups, as appropriate. Multivariate Cox regression analysis was used for PFS and OS. Statistical significance was set at a two-sided *P*-value < 0.05. All statistical analyses were performed using Stata 16.0 software (Stata Corp LP, College Station, TX, USA).

## Results

### Patient characteristics

A total of 445 patients with LA-HNSCC were enrolled in this study and analyzed retrospectively. The median age was 61 years (range, 24–89), and 385 (87%) were male. The primary tumor location was the oropharynx in 191 (43%) of the cases, followed by oral cavity in 106 (24%), hypopharynx in 64 (14%), larynx in 57 (13%), and other sites in 27 (6%). Other sites included maxillary sinus, nasal cavity, ethmoid sinus, and unknown primary squamous carcinoma. The most common clinical tumor (T) and lymph node (N) stage was T2 in 172 (39%) and N2 in 245 (55%), respectively. About 58% (256) of study patients was unknown for HPV infection. Of 189 patients who were tested for HPV status, 48% (90/189) were positive. Table 1 summarized the demographics of study population.

**Table 1:** Baseline characteristics of locally advanced head & neck squamous cell carcinoma

Characteristics	Treatment Strategy		***p***-value*		
	CCRT group ***n*** = 229 (51.5%)	Surgery group ***n*** = 187 (42.0%)		Inadequate Tx ***n*** = 29 (6.5%)	Total ***N*** = 445 (100%)
Age, median [range], years	61 [30–81]	60 [24–89]	0.633	67 [36–82]	61 [24–89]
Gender			0.125		
Female	25 (41.7%)	30 (50.0%)		5 (8.3%)	60 (100%)
Male	204 (53.0%)	157 (40.8%)		24 (6.2%)	385 (100%)
ECOG PS			< 0.001		
0	40 (72.7%)	9 (16.4%)		6 (10.9%)	55 (100%)
1	153 (71.2%)	47 (21.9%)		15 (7.0%)	215 (100%)
2	12 (70.6%)	3 (17.7%)		2 (11.8%)	17 (100%)
3	4 (100%)	0 (0.0%)		0 (0.0%)	4 (100%)
Unknown	20 (13.0%)	128 (83.1%)		6 (3.9%)	154 (100%)
Smoking history			< 0.001		
Never	37 (37.4%)	54 (54.6%)		8 (8.1%)	99 (100%)
Former	62 (46.3%)	62 (46.3%)		10 (7.5%)	134 (100%)
Current	53 (49.5%)	46 (43.0%)		8 (7.5%)	107 (100%)
Unknown	77 (73.3%)	25 (23.8%)		3 (2.9%)	105 (100%)
Alcohol history			< 0.001		
Do not drink	49 (40.8%)	60 (50.0%)		11 (9.2%)	120 (100%)
Drink alcohol	71 (43.6%)	83 (50.9%)		9 (5.5%)	163 (100%)
Unknown	109 (67.3%)	44 (27.2%)		9 (5.6%)	162 (100%)
Primary tumor location			< 0.001		
Oropharynx	105 (55.0%)	73 (38.2%)		13 (6.8%)	191 (100%)
Oral cavity	30 (28.3%)	70 (66.0%)		6 (5.7%)	106 (100%)
Hypopharynx	42 (65.5%)	16 (25.0%)		6 (9.4%)	64 (100%)
Larynx	31 (54.4%)	24 (42.1%)		2 (3.5%)	57 (100%)
Others	21 (77.8%)	4 (14.8%)		2 (7.4%)	27 (100%)
Histologic grade			< 0.001		
Well differentiated	22 (32.4%)	41 (60.3%)		5 (7.4%)	68 (100%)
Moderate differentiated	58 (36.5%)	97 (61.0%)		4 (2.5%)	159 (100%)
Poorly differentiated	42 (63.6%)	21 (31.8%)		3 (4.6%)	66 (100%)
Not assessed	107 (70.4%)	28 (18.8%)		17 (11.4%)	152 (100%)
T classification			< 0.001		
T1	21 (31.8%)	43 (65.2%)		2 (3.0%)	66 (100%)
T2	85 (49.4%)	80 (46.5%)		7 (4.1%)	172 (100%)
T3	57 (60.6%)	30 (31.9%)		7 (7.5%)	94 (100%)
T4a / T4b	51 / 14 (58.6%)	31 / 2 (29.7%)		11 / 2 (11.7%)	93 / 18 (100%)
Unknown	1 (50.0%)	1 (50.0%)		0 (0.0%)	2 (100%)
N classification			< 0.001		
N0	25 (48.1%)	24 (46.2%)		3 (5.8%)	52 (100%)
N1	56 (40.0%)	79 (56.4%)		5 (3.6%)	140 (100%)
N2	142 (58.0%)	83 (33.9%)		20 (8.2%)	245 (100%)
N3	6 (85.7%)	1 (14.3%)		0 (0.0%)	7 (100%)
Unknown	0 (0.0%)	0 (0.0%)		1 (100%)	1 (100%)
P16/HPV status			< 0.001		
Negative	16 (16.2%)	79 (79.8%)		4 (4.0%)	99 (100%)
Positive	45 (50.0%)	43 (47.8%)		2 (2.2%)	90 (100%)
Unknown	168 (65.6%)	65 (25.4%)		23 (9.0%)	256 (100%)

*p*-value was calculated by t-test or Chi-square test as appropriate between CCRT and surgery group

*PS* Performance status, *HPV* Human papillomavirus

### Treatment strategy

**Fig. 1:**
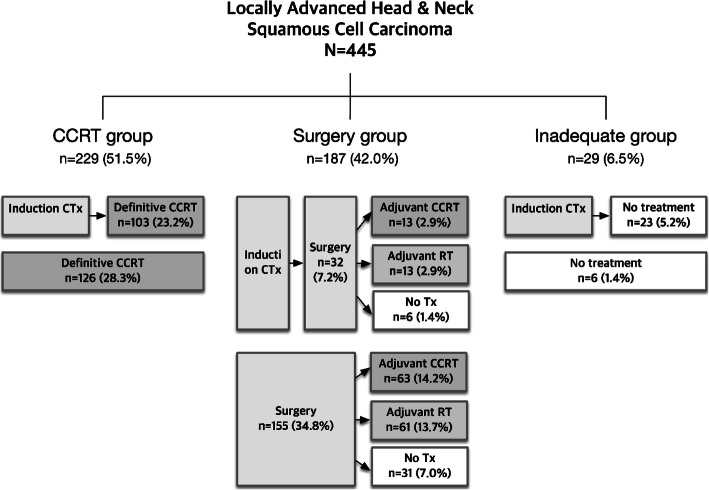
Flowchart for the treatment of locally advanced head & neck squamous cell carcinoma (*N* = 445). CCRT, concurrent chemoradiotherapy; CTx, chemotherapy; RT, radiotherapy; Tx, treatment

Based on treatment intents, patients received definitive CCRT in 229 (52%) of cases and surgery in 187 (42%). The remaining 29 (7%) did not receive adequate treatment. Approximately 158 (36%) of the study population received IC. In 229 patients from the CCRT group, 45% (103 patients) underwent IC prior to definitive CCRT. In 187 patients from the surgery group, 17% (32 patients) received IC followed by surgery with curative intent. Of the 29 patients in the inadequate treatment group, about 80% (23/29) failed to receive subsequent treatment after IC. Taken together, 385 (87%) of the patients were treated with combined treatment modalities (Fig. 1).

### Treatment characteristics

Details of the treatment modalities are shown in Table 2. For 158 patients receiving IC, the preferred regimen was DP (docetaxel and cisplatin) in 49% (77/158) of the patients, TPF (docetaxel, cisplatin and fluorouracil) in 27% (42/158), FP (Fluorouracil and cisplatin) in 18% (28/158), and other therapies in 7% (11/158). The median number of cycles for chemotherapy was 3 (range 1–5). The best overall response was a complete response (CR) in 16% (25/158), a partial response (PR) in 55% (87/158), stable disease (SD) in 20% (31/158) and progressive disease (PD) in 10% (15/158) of the patients. Patients presenting a good performance status were more likely to receive IC compared with those with a poor performance status (*p* <  0.001). For oropharyngeal and hypopharyngeal cancer, patients received IC more frequently compared with those in the oral cavity and larynx group (*p* <  0.001). For clinical T and N classification, patients presenting advanced stage T and N were more likely to receive IC (*p* < 0.001, Supplementary Table 1).

**Table 2:** Characteristics of treatment modalities in patients with LA-HNSCC

Treatment		Number (%)
**Induction chemotherapy**		*n = 158*
Regimen	Docetaxel + Cisplatin	77 (48.7%)
	Docetaxel + Cisplatin + Fluorouracil	42 (26.6%)
	Fluorouracil + Cisplatin	28 (17.7%)
	Others	11 (7.0%)
Number of cycles	Median: 3 cycles	Range 1–5
Best overall response	Complete response	25 (15.8%)
	Partial response	87 (55.1%)
	Stable disease	31 (19.6%)
	Progressive disease	15 (9.5%)
**Definitive Concurrent chemoradiotherapy (CCRT)**		*n = 229*
CCRT regimen	Weekly cisplatin	133 (58.1%)
	3-weekly cisplatin	63 (27.5%)
	Fluorouracil + Cisplatin	22 (9.6%)
	Others	11 (4.8%)
Total radiation dose (Gy)	Mean: 62.5 / Median: 67.5 Gy	Range 32–72
Best overall response	Complete response	148 (65.2%)
	Partial response	42 (18.5%)
	Stable disease	21 (9.3%)
	Progressive disease	16 (7.1%)

Of the 305 patients receiving CCRT, the goal was to treat 75% (229/305) with definitive and 24% (76/305) with adjuvant therapy. The preferred regimen for definitive CCRT was weekly cisplatin for 58%, 3-weekly cisplatin for 28%, and 5-fluorouracil and cisplatin for 10% of the patients. Cetuximab was selected for only 3% of the patients. The median dose of irradiation was 67.5Gy (range 32–72). The best overall response was a CR in 65%, PR in 19%, SD in 9%, and PD in 7%. The CCRT regimen was not different between the definitive and adjuvant setting (*p* = 0.151, *unpublished data*).

### Study outcomes

With a median follow-up period of 39.3 months (95% CI 35.4–43.1), 113 deaths were observed. For 445 patients, 1-year and 2-year survival rates were 88.7% (95% CI 85.2–91.3) and 79.8% (95% CI 75.4–83.4), respectively. A median OS was not reached. When drawing a flowchart with respect to treatment intent, 52% (229/445) of the patients received definitive CCRT, and 42% (187/445) underwent surgery. The most frequently adopted treatment strategy was definitive CCRT in 28% (126/445), IC followed by definitive CCRT in 23% (103/445), and surgery followed by adjuvant CCRT or radiotherapy in 14% (63/445) (Fig. 1).

**Fig. 2:**
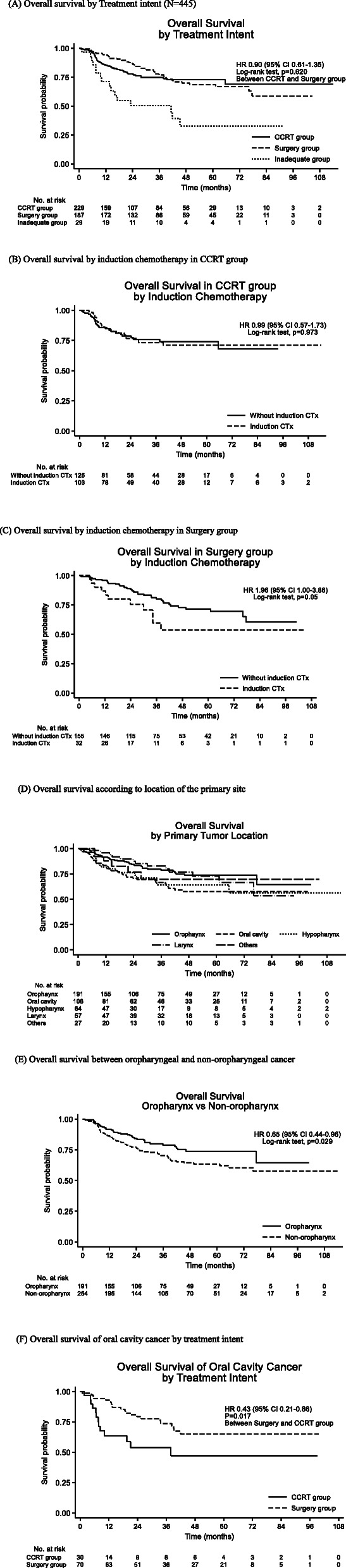
**a** Overall survival by Treatment intent (*N* = 445). **b** Overall survival by induction chemotherapy in CCRT group. **c** Overall survival by induction chemotherapy in Surgery group. **d** Overall survival according to location of the primary site. **e** Overall survival between oropharyngeal and non-oropharyngeal cancer. **f** Overall survival of oral cavity cancer by treatment intent

When comparing survival probabilities between the CCRT and surgery groups, OS was not significantly different (HR 0.90; 95% CI 0.61–1.35; *p* = 0.620) (Fig. 2a). When patients failed to receive adequate treatment following IC or refused anticancer treatment, OS was the poorest (Fig. 2a). To evaluate the clinical role of IC, we analyzed the prognostic impact of IC in the CCRT and surgery groups. In the CCRT group, survival probabilities were not significantly different by administration of IC (HR 0.99; 95% 0.57–1.73; *p* = 0.973) (Fig. 2b). In the surgery group, however, patients receiving IC prior to surgery exhibited inferior OS (HR, 1.96; 95% CI, 1.00–3.86; *p* = 0.05) (Fig. 2c). After adjustment of covariate, the estimates of IC in surgery group was not statistically significant (HR 1.48; 95% CI 0.58–3.82; *p* = 0.423). According to the primary tumor location, patients with oropharyngeal cancer showed better survival probability than non-oropharyngeal cancer (HR 0.65; 95% CI 0.44–0.96, *p* = 0.029) (Fig. 2d, e). Compared with other primary tumor locations, oral cavity cancer showed the worst survival outcome (Fig. 2D). In oral cavity cancer, the surgical approach exhibited better survival probability than CCRT (HR 0.43; 95% CI 0.21–0.86; *p* = 0.017) (Fig. 2f).

### Multivariate analyses for PFS and OS

Multivariate analyses for PFS revealed that primary tumor location of other sites (maxillary sinus, nasal cavity, ethmoid sinus, and unknown primary squamous carcinoma versus oropharyngeal cancer), advanced T classification (from one unit to the next), and inadequate treatment (vs. CCRT) were significant predictors for PFS (Table 3, Supplementary Table 2).

**Table 3:** Patients and tumor characteristics related to progression-free survival and overall survival according to multivariate analysis

Outcomes		Estimate (95% CI)	***p***-value
**Progression-free survival**		**Hazard ratio**	
Primary location (Others vs. Oropharynx)		3.65 (1.77–7.52)	< 0.001
T classification (from one unit to next)		1.43 (1.09–1.89)	0.011
Treatment strategy (Inadequate vs. CCRT)		2.28 (1.23–4.20)	0.009
**Overall survival**			
HPV status (Positive vs. Negative)		0.29 (0.14–0.63)	0.002
Primary location (Oral cavity vs. Oropharynx)		1.82 (1.22–2.71)	0.003
T classification (from one unit to next)		1.30 (1.08–1.57)	0.006
N classification (from one unit to next)		1.58 (1.20–2.08)	0.001
Treatment strategy (Inadequate vs. CCRT)		2.31 (1.35–3.97)	0.002

*CCRT* Concurrent chemoradiotherapy, *HPV* Human papillomavirus

With respect to mortality, HPV positivity (vs. negative) was an independent prognostic indicator for improved survival. Primary tumor location in the oral cavity (vs. oropharynx), advanced T and N classification, and inadequate treatment (vs. CCRT) were independent predictors for poor survival (Table 3, Supplementary Table 3).

## Discussion

This nationwide retrospective cohort study including 445 patients with LA-HNSCC found that 87% of the patients received multimodality treatment modalities. Based on treatment intents, 52% of the patients received definitive CCRT, while 42% underwent surgery. Approximately 36% of the study population received IC. Regarding multidisciplinary approaches, the preferred treatment strategy was definitive CCRT in 28%, IC then definitive CCRT in 23%, surgery followed by adjuvant CCRT in 14% or adjuvant radiotherapy in 14% of the patients. Overall outcomes for one- and two-year survival rates were 88.7 and 79.8%, respectively.

In the context of LA-HNSCC therapeutics, our study provides valuable information for drawing a general treatment landscape. OS was not different between definitive the CCRT and surgery groups. Given that IC was administered in approximately one-third of our patients with more advanced disease, IC did not show survival advantages in either the CCRT or surgery group. Though a recommendation for IC, except for the purpose of laryngeal preservation, has yet to be established, [7–9] only 19% of our patients with laryngeal cancer received IC. In other words, the real-world practice indicated that IC was being performed more actively for advanced stages of LA-HNSCC other than laryngeal cancer without definite evidence of its survival advantages. The patients receiving IC prior to surgery showed poorer OS than the patients receiving surgery without IC. The reason is that IC was performed when the tumor is bulky, and node is advanced (Supplementary Table 1). Approximately 23 patients (5%) recognized in Fig. 1 did not receive subsequent definitive treatment after IC and showed the worst OS (Fig. 2a). Residual toxicity following IC could complicate succeeding definitive treatments, especially surgery, so physicians need to be more cautious in selecting the sequence of treatment modalities. In oral cavity cancer, which had the worst survival outcome, the surgical approach showed survival benefits over CCRT in our study. These results provide us valuable insights to build the optimal treatment strategy in oral cavity cancer.

Based on the TAX-323/EORTC-24971 and TAX-324 phase III trials, the TPF regimen as IC is now accepted to be an evidence-based regimen of choice [10–12]. This is because the TPF regimen proved clear survival benefits over FP chemotherapy in unresectable LA-HNSCC [13]. Regarding toxicities, almost 80% of the patients treated with TPF regimen experience grade 3–4 neutropenia and 12% developed infection. Poor compliance (about 75% of patients completed the protocol) due to toxicities was another concern for the TPF regimen. In our study, DP was the most frequently administered regimen. For toxicity and adherence concerns, DP may be considered the preferred regimen in Korea instead of TPF [14, 15]. Given that there is currently no direct study comparing outcomes the DP and the TPF regimens, further research regarding optimal IC regimens is needed.

CCRT with cisplatin remains the gold standard for the treatment of LA-HNSCC [16]. In our LA-HNSCC population, definitive CCRT was the main therapeutic modality for more than half (52%) of the patients. Regarding the schedule of cisplatin during definitive CCRT, weekly cisplatin was used approximately two times more frequently than 3-weekly schedule (58% vs. 28%). In a recent meta-analysis, Szturz et al. found that both high- and low-dose cisplatin regimens yield similar survival outcomes for postoperative and definitive CCRT [17]. This finding is consistent with a population based study of US military veterans that included over 2900 patients [18]. Given that 3-weekly cisplatin was associated with significantly more toxicity than weekly -cisplatin, tolerability is a key factor in selection. Preference for weekly cisplatin in our study reflects the physicians’ tendency to value safety [19, 20]. For postoperative CCRT in high-risk disease, a recent phase III study, conducted at a single institution in India, demonstrated that two-year locoregional control was superior in patients receiving 100 mg/m^2^ cisplatin every 3 weeks compared with 30 mg/m^2^ cisplatin weekly (73.1% vs. 58.5%, *p* = 0.014) [6]. Because 3-weekly cisplatin results in more toxicity than weekly cisplatin, physicians need to choose a treatment regimen that balances efficacy with toxicity.

Our multivariate analyses demonstrated that positive HPV status was a good independent prognostic factor, consistent with other studies [21–23]. However, these results should be interpreted carefully, because the status of HPV infection was tested for only 43% of the patients and the prognostic role of HPV status in non-oropharyngeal cancer is inconclusive [24]. In particular, oral cavity cancer conferred the worst survival. Therefore, special attention to improve outcome in oral cavity cancer is warranted.

Several limitations to our study need to be addressed. First, data for this outcome study was collected and analyzed retrospectively, which has inherent selection bias. However, a relatively large number of LA-HNSCC patients (*n* = 445) were evaluated from thirteen nationwide referral hospitals, which represented a real-world situation in Korea. It will certainly be considered that the number of patients in our study is not sufficient to draw a definitive conclusion. Second, heterogeneous patients with tumor arising from various sites received different therapeutics. This limits the accurate interpretation of the study results. Lastly, our study could not collect toxicity profiles due to the risk of underestimating the retrospectively collected data.

## Conclusions

Most patients with LA-HNSCC were treated with combined multidisciplinary therapeutics and showed favorable survival outcomes. Definitive CCRT, IC then definitive CCRT, and surgery followed by adjuvant CCRT or radiotherapy are the preferred multidisciplinary strategies. Though one-third of the patients received IC, its clinical role should be further evaluated in clinical trials. Our results are essential to understanding the patterns of multidisciplinary team approaches in real-world practice and to provide valuable insights regarding optimal therapeutic strategies for physicians. Prospective data is still needed to better assess therapy modalities in LA-HNSCC.

## Supplementary information

**Additional file 1: Figure S1.** Overall survival according to each treatment. **Table S1.** Demographics by receiving induction chemotherapy. **Table S2.** Univariate and multivariate analyses for progression-free survival in 445 evaluable patients with LA-HNSCC. **Table S3.** Univariate and multivariate analyses for overall survival 445 evaluable patients with LA-HNSCC.

## Data Availability

Data would be available from the corresponding author on reasonable request.

## Abbreviations

HNSCC: Head and neck squamous cell carcinoma
LA: Locally advanced
CCRT: Chemoradiotherapy;
IC: Induction chemotherapy
CT: Computed tomography
MRI: Magnetic Resonance Imaging
PFS: Progression-free survival
OS: Overall survival
CR: Complete response
PR: Partial response
PD: Progressive disease
HPV: Human papilloma virus
