# Supplementary material for: Treatment strategy and outcomes in locally advanced head and neck squamous cell carcinoma: a nationwide retrospective cohort study (KCSG HN13–01)
Source: BMC Cancer. 2020 Aug 27;20:813. doi: 10.1186/s12885-020-07297-z (PMC7450571; doi:10.1186/s12885-020-07297-z)
Supplement: Supplementary file 1 — Additional file 1: Figure S1. Overall survival according to each treatment. Table S1. Demographics by receiving induction chemotherapy. Table S2. Univariate and multivariate analyses for progression-free survival in 445 evaluable patients with LA-HNSCC. Table S3. Univariate and multivariate analyses for overall survival 445 evaluable patients with LA-HNSCC. [file 12885_2020_7297_MOESM1_ESM.docx]

**Supplementary Table 1**: Demographics by receiving induction chemotherapy

| **Characteristics** | **Total**  **N=445** | | **Induction chemotherapy** | | |  |
| --- | --- | --- | --- | --- | --- | --- |
|  |  |  | **Not received, n=287** | | **Received, n=158** | ***p*-value** |
| Age, median (range), years | 61 (24–88) | | 61 (24–88) | | 61 (24–88) | 0.945 |
| Gender  Female  Male | 60 (13%)  385 (87%) | | 37 (13%)  250 (87%) | | 23 (15%)  135 (85%) | 0.623 |
| ECOG PS  0  1  2  3  Unknown | 55 (12%)  215 (48%)  17 (4%)  4 (1%)  154 (35%) | | 20 (7%)  111 (39%)  9 (3%)  4 (1%)  143 (50%) | | 35 (22%)  104 (66%)  8 (5%)  0 (0%)  11 (7%) | <0.001 |
| Smoking history  Never  Former  Current  Unknown | 99 (22%)  134 (30%)  107 (24%)  105 (24%) | | 70 (24%)  97 (34%)  78 (27%)  42 (15%) | | 29 (18%)  37 (23%)  29 (18%)  63 (40%) | <0.001 |
| Alcohol history  Do not drink  Drink alcohol  Unknown | 120 (27%)  163 (37%)  162 (36%) | | 87 (30%)  126 (44%)  74 (26%) | | 33 (21%)  37 (23%)  88 (56%) | <0.001 |
| Primary tumor location  Oropharynx  Oral cavity  Hypopharynx  Larynx  Others | 191 (43%)  106 (24%)  64 (14%)  57 (13%)  27 (6%) | | 108 (38%)  85 (30%)  32 (11%)  46 (16%)  16 (6%) | | 83 (53%)  21 (13%)  32 (20%)  11 (7%)  11 (7%) | <0.001 |
| Histologic grade  Well differentiated  Moderate differentiated  Poorly differentiated  Not assessed | 68 (15%)  159 (36%)  66 (15%)  152 (34%) | | 45 (16%)  118 (41%)  42 (15%)  82 (29%) | | 23 (15%)  41 (26%)  24 (15%)  70 (44%) | 0.006 |
| T classification  T1  T2  T3  T4a / T4b  Unknown | 66 (15%)  172 (39%)  94 (21%)  93 / 18 (25%)  2 (<1%) | | 53 (18%)  121 (42%)  61 (21%)  47 / 5 (18%)  0 (0%) | | 13 (8%)  51 (32%)  33 (21%)  46 / 13 (37%)  2 (1%) | <0.001 |
| N classification  N0  N1  N2  N3  Unknown | 52 (12%)  140 (31%)  245 (55%)  7 (2%)  1 (<1%) | | 36 (13%)  118 (41%)  128 (45%)  5 (2%)  0 (0%) | | 16 (10%)  22 (14%)  117 (74%)  2 (1%)  1 (1%) | <0.001 |
| P16/HPV status  Negative  Positive  Unknown | 99 (22%)  90 (20%)  256 (58%) | | 77 (27%)  62 (22%)  148 (52%) | | 22 (14%)  28 (18%)  108 (68%) | 0.001 |
| PS, performance status; HPV, human papillomavirus | |  | |  | |  |

**Supplementary table 2**: Univariate and multivariate analyses for progression-free survival in 445 evaluable patients with LA-HNSCC

| **Characteristics** | **Univariate** | | **Multivariate** | | |
| --- | --- | --- | --- | --- | --- |
|  | **Hazards ratio (95% CI)** | ***P*** | **Hazards ratio (95% CI)** | ***P*** |  |
| Age, years  > 60 vs. ≤ 60 | 1.21 (0.76 – 2.12) | 0.355 |  |  |  |
| Gender  Male vs. female | 1.20 (0.54 – 2.64) | 0.653 |  |  |  |
| ECOG PS  2-3 vs. 0-1 | 2.39 (1.01 – 5.62) | 0.046 |  |  |  |
| Smoking history  Current or former vs. never | 0.92 (0.52 – 1.64) | 0.783 |  |  |  |
| Alcohol history  Drink vs. Do not drink | 1.45 (0.81 – 2.60) | 0.211 |  |  |  |
| HPV status  Positive vs. Negative | 0.45 (0.14 – 1.45) | 0.177 |  |  |  |
| Primary tumor location  Oropharynx  Oral cavity  Hypopharynx  Larynx  Others | 1 (reference)  0.81 (0.38 – 1.73)  1.05 (0.44 – 2.49)  1.64 (0.79 – 3.40)  4.33 (2.08 – 9.01) | 0.588  0.912  0.185  <0.001 | 1 (reference)  3.65 (1.77 – 7.52) | <0.001 |  |
| T classification | 1.56 (1.20 – 2.02) | 0.001 | 1.43 (1.09 – 1.89) | 0.011 |  |
| N classification | 1.46 (0.99 – 2.15) | 0.055 |  |  |  |
| Induction chemotherapy  Yes vs. No | 1.11 (0.66 – 1.89) | 0.692 |  |  |  |
| Treatment strategy  CCRT  Surgery  Inadequate | 1 (reference)  0.92 (0.54 – 1.56)  1.24 (0.44 – 3.54) | 0.758  0.683 | 1 (reference)  2.28 (1.23 – 4.20) | 0.009 |  |
| * Reference, HPV: human papilloma virus; PS: performance status | | | | | |

**Supplementary table 3**: Univariate and multivariate analyses for overall survival 445 evaluable patients with LA-HNSCC

| **Characteristics** | **Univariate** | | **Multivariate** | | |
| --- | --- | --- | --- | --- | --- |
|  | **Hazards ratio (95% CI)** | ***P*** | **Hazards ratio (95% CI)** | ***P*** |  |
| Age, years  > 60 vs. ≤ 60 | 1.47 (1.01 – 2.13) | 0.045 |  |  |  |
| Gender  Male vs. female | 1.02 (0.59 – 1.76) | 0.938 |  |  |  |
| ECOG PS  2-3 vs. 0-1 | 1.95 (0.98 – 3.91) | 0.059 |  |  |  |
| Smoking history  Current or former vs. never | 01.10 (0.70 – 1.73) | 0.673 |  |  |  |
| Alcohol history  Drink vs. Do not drink | 1.23 (0.76 – 1.97) | 0.399 |  |  |  |
| HPV status  Positive vs. Negative | 0.25 (0.11 – 0.56) | 0.001 | 0.29 (0.14 – 0.63) | 0.002 |  |
| Primary tumor location  Oropharynx  Oral cavity  Hypopharynx  Larynx  Others | 1 (reference)  1.82 (1.16 – 2.87)  1.73 (0.99 – 3.01)  1.05 (0.56 – 1.97)  1.21 (0.51 – 2.86) | 0.09  0.053  0.888  0.670 | 1 (reference)  1.82 (1.22 – 2.71) | 0.003 |  |
| T classification | 1.39 (1.15 – 1.67) | <0.001 | 1.30 (1.08 – 1.57) | 0.006 |  |
| N classification | 1.49 (1.13 – 1.98) | 0.005 | 1.58 (1.20 – 2.08) | 0.001 |  |
| Induction chemotherapy  Yes vs. No | 1.48 (1.02 – 2.15) | 0.039 |  |  |  |
| Treatment strategy  CCRT  Surgery  Inadequate | 1 (reference)  0.90 (0.61 – 1.35)  2.78 (1.58 – 4.87) | 0.620  <0.001 | 1 (reference)  2.31 (1.35 – 3.97) | 0.002 |  |
| * Reference, HPV: human papilloma virus; PS: performance status | | | | | |

Supplementary Figure 1. Overall survival according to each treatment
